# Automatic Prediction of Rheumatoid Arthritis Disease Activity from the Electronic Medical Records

**DOI:** 10.1371/journal.pone.0069932

**Published:** 2013-08-16

**Authors:** Chen Lin, Elizabeth W. Karlson, Helena Canhao, Timothy A. Miller, Dmitriy Dligach, Pei Jun Chen, Raul Natanael Guzman Perez, Yuanyan Shen, Michael E. Weinblatt, Nancy A. Shadick, Robert M. Plenge, Guergana K. Savova

**Affiliations:** 1 Informatics Program, Boston Children's Hospital, Boston, Massachusetts, United States of America; 2 Rheumatology and Immunology Department, Brigham and Women's Hospital, Boston, Massachusetts, United States of America; 3 Harvard Medical School, Harvard University, Boston, Massachusetts, United States of America; 4 Rheumatology Research Unit, Instituto de Medicina Molecular, Faculdade de Medicina da Universidade de Lisboa, Lisbon, Portugal; 5 School of Public Health, Harvard University, Boston, Massachusetts, United States of America; Wayne State University, United States of America

## Abstract

**Objective:**

We aimed to mine the data in the Electronic Medical Record to automatically discover patients' Rheumatoid Arthritis disease activity at discrete rheumatology clinic visits. We cast the problem as a document classification task where the feature space includes concepts from the clinical narrative and lab values as stored in the Electronic Medical Record.

**Materials and Methods:**

The Training Set consisted of 2792 clinical notes and associated lab values. Test Set 1 included 1749 clinical notes and associated lab values. Test Set 2 included 344 clinical notes for which there were no associated lab values. The Apache clinical Text Analysis and Knowledge Extraction System was used to analyze the text and transform it into informative features to be combined with relevant lab values.

**Results:**

Experiments over a range of machine learning algorithms and features were conducted. The best performing combination was linear kernel Support Vector Machines with Unified Medical Language System Concept Unique Identifier features with feature selection and lab values. The Area Under the Receiver Operating Characteristic Curve (AUC) is 0.831 (σ = 0.0317), statistically significant as compared to two baselines (AUC = 0.758, σ = 0.0291). Algorithms demonstrated superior performance on cases clinically defined as extreme categories of disease activity (Remission and High) compared to those defined as intermediate categories (Moderate and Low) and included laboratory data on inflammatory markers.

**Conclusion:**

Automatic Rheumatoid Arthritis disease activity discovery from Electronic Medical Record data is a learnable task approximating human performance. As a result, this approach might have several research applications, such as the identification of patients for genome-wide pharmacogenetic studies that require large sample sizes with precise definitions of disease activity and response to therapies.

## Introduction

Long-term outcome in patients with rheumatoid arthritis (RA) is highly dependent upon an aggressive pharmacological control of inflammation early in the disease course. Despite the importance of selecting the optimal medication soon after disease onset, there is no reliable biomarker predictor of drug treatment response. As a consequence, RA patients often suffer irreversible joint destruction while a physician searches for an effective drug. Disease activity modifying anti-rheumatic drugs (DMARDS) are considered first-line therapy for RA while new biologic agents, such as drugs that block the inflammatory cytokine TNF-alpha are considered highly effective yet induce remission in only 30% of patients [1], [2], [3], [4], [5]. The choice of drug therapy is based on disease activity levels and clinical prognostic features. A genetic biomarker that associates with high likelihood of biologic agent response could change this paradigm, and improve outcomes in early RA.

Disease activity assessed at clinical visits drives the choice of therapy. Standardized disease activity levels are measured at regular intervals as the primary endpoint in RA clinical trials. However, defining disease activity before and after drug exposure in observational Electronic Medical Record (EMR) data is challenging, as clinicians typically do not regularly code disease activity in structured fields but describe it as free text in the clinical narrative. For example, at our institution, we have a structured disease activity tool [6] and a longitudinal cohort study [7] that collect disease activity data at individual patient visits, but these structured data are available on a minority of visits (20–30%).

One example of a structured tool used by Partners HealthCare is the Disease Activity Score in 28 joints (DAS28) tool scored by study rheumatologists for RA patients followed annually in a cohort study, the Brigham&Womens Rheumatoid Arthritis Sequential Study (BRASS), and clinical rheumatologists for RA patients. DAS28 is a composite index developed and validated for use in clinical trials. It is based on weighted variables for swollen joint count, tender joint count, the C-reactive protein level (CRP) or erythrocyte sedimentation rate (ESR), and patient-reported assessment of global health. The original DAS algorithm was developed from clinical and laboratory variables assessed by six rheumatologists in a prospective study of three years' duration. They defined high, moderate, low and remission disease activity based on associations with changes in medication [8]. Once the DAS algorithm was developed, it was validated in additional RA patients [9], and eventually applied to thousands of patients in clinical trials, patient registries and routine office visits. Remarkably, the original analysis was performed in only 113 RA patients in the 1980's, prior to the introduction of biological DMARDs; nonetheless, the essential components of the algorithm are in use today.

However, the majority of the disease activity information is not created through structured tools; rather, it is scattered as free text descriptions throughout the clinical narrative within the EMR. Over the past decade, many natural language processing (NLP) systems have been utilized in various types of healthcare EMR applications [10], [11], [12], [13], [14], [15], [16], [17] to process the clinical narrative and extract relevant information from it. There are tools built for specific tasks such as *SymText* utilized in identifying pneumonia-related concepts and finding pneumonia-supported reports [13], [14]. The Unified Medical Language System (UMLS) [18] is frequently used as a source of ontology codes, for example the terms *rheumatoid arthritis* and *RA* are assigned the same UMLS concept unique identifier (CUI) C0003873 with a semantic type of Disease/Disorders. The UMLS provides CUIs for over 130 biomedical ontologies.

For machine learning purposes, the clinical narrative is typically represented as a vector of features, where the features can be such as expert-provided terms related to a target disease [16], all distinct terms (bag-of-words (BOW) [19]) or UMLS concepts [17] found in a clinical document. A disadvantage of the task-specific dictionaries is that they are manually tailored by domain experts in a time-consuming process. While these features have proven helpful [8] [15] , they might not be exhaustive. On the other hand, the drawback of using all unique terms is that the feature space becomes very big. A small corpus of clinical narratives may have a representation of thousands of features. Therefore, different methods for statistical feature selection to reduce the feature space [20], [21] have been proposed. A range of feature selection methods are summarized by Joachims [22], Ma & Huang [23], Sayes, Inza, & Larranaga [24], Zhao et al. [25], and Yang et al. [26].

In this study, we aim to develop methods to automatically discover RA disease activity at discrete rheumatology clinic visits based on EMR data. Such an automated method has the potential to speed up the collection of patient cohorts from the EMR for further clinical investigation, currently a time-consuming manual process. We approach the problem as a classification task. NLP technologies are utilized to analyze the EMR clinical text and transform it into computable features. In our previous work [27], [28], we (1) explored multiple feature representations of clinical notes such as user-defined terms, UMLS CUIs [18], BOW, and word-CUI bigrams, and (2) tested several filter-based feature selection methods to reduce the dimensionality of the feature space and improve classification. In this manuscript, our goal is to build on that work and to investigate algorithms for discovering disease activity level using EMR data. This work is the first step for future studies of pharmacogenetic predictors of biologic agent drug response in large cohort studies harvested from big data EMRs.

All abbreviations used in this paper are listed in Table S6.

## Materials and Methods

### Materials

The RA EMR cohort used in this study included 5,900 patients from Partners HealthCare RA case status was assigned based on a validated algorithm developed at Partners HealthCare that used a combination of variables extracted from the clinical narrative and codified EMR data to automatically discover RA cases [15]. The EMR algorithm has a 0.94 positive predictive value (PPV) for RA diagnosis with demonstrated portability across two other EMRs [29]. We also devised a series of filtering criteria to select informative notes from rheumatology clinic visits from the cohort, excluding educational notes, telephone notes, and visits to the infusion center, primary care, or other subspecialists (Consult the Filtering Criteria S1 for a list of the filtering criteria). Based on recommended thresholds in clinical trials [30], DAS28 score was categorized into High (DAS28>5.1), Moderate (DAS28>3.2–5.1), Low (DAS28≥2.6–3.2), and Remission (DAS28<2.6). We used the four DAS28-derived categories of disease activity as gold standard labels for the Training Set and Test Set 1 described below. Lab values were retrieved from a structured EMR database separate from the database containing the text blob of the clinical narrative.

Among the RA EMR Cohort, disease activity was quantitatively measured in 852 RA patients enrolled in longitudinal cohort study, the BRASS. We selected 2792 notes from visits at rheumatology clinics from these 852 patients to form the Training Set. Each note has a DAS28 score and associated CRP and/or ESR lab values, and MD-estimated DAS scored at the time of the visit (without laboratory data available). The disease activity labels associated with each clinical note were automatically assigned by using the DAS28 score into High, Moderate, Low, or Remission categories.

Among the RA EMR cohort, disease activity was quantitatively measured using an online disease activity tool for an independent group of 821 RA patients as part of clinical care at Brigham & Women's Hospital. We selected 1749 notes from rheumatology visits from these 821 patients to form Test Set 1. Each note has a DAS28 score and associated CRP and/or ESR values, and MD-estimated DAS scored at the time of the visit (without laboratory data available). The disease activity labels associated with each document were automatically assigned by using the DAS28 score into High, Moderate, Low, or Remission categories following the same procedure as for the Training Set. To measure the inter-annotator agreement (IAA) as *F1 score* [31], two domain experts reviewed 93 of these clinical notes to classify disease activity into the four disease activity categories, without knowledge of laboratory values.

We randomly selected 445 clinical notes for a third group of 445 RA patients (one note per each patient) without structured DAS28 from the remaining RA EMR cohort to form an independent test set comprised of notes from regular care to form Test Set 2. Three domain experts (study rheumatologists) independently reviewed these notes to assign clinical disease activity labels (High, Moderate, Low and Remission) based on clinical data in the notes alone with no additional outside lab values since CRP results were not available to the clinician at the time of the visit. Disagreements were resolved in an adjudication step. The IAA for Moderate and Low categories was consistently low with difficulty reaching consensus. Thus, reviewers subsequently labeled disease activity into aggregate Moderate/High or Low/Remission categories. Some of the notes did not contain enough information for the domain experts to make a reliable classification, therefore they were removed. Thus, the final Test Set 2 included 344 notes for 344 RA patients. Test Set 2 is used to test the portability of the methods for automatic disease activity labeling of notes without CRP/ESR laboratory data.

Table 1 presents the dataset characteristics.

**Table 1:** Dataset characteristics.

	*Training Set*	*Test Set 1*	*Test Set 2*
High Disease Activity	506 notes	190 notes	
Moderate Disease Activity	966 notes	610 notes	
Aggregate High/Moderate Disease Activity	1472 notes	800 notes	133 notes
Low Disease Activity	369 notes	312 notes	
Remission Disease Activity	951 notes	637 notes	
Aggregate Low/Remission Disease Activity	1320 notes	949 notes	211 notes
Total	2792 notes	1749 notes	344 notes
Agreement	MD/DAS28: 0.81	MD/DAS28: 0.87	Inter-annotator agreement: 0.87

The study was conducted under an approved Institutional Review Board (IRB) protocol.

### Methods

**Figure 1:**
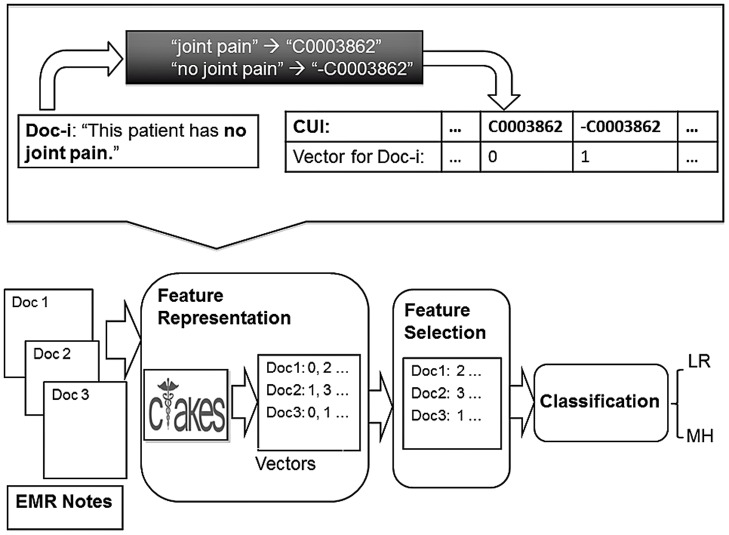
Representation of the processing flow for automatic disease activity labeling. Abbreviations: CUI – Unified Medical Language System Concept Unique Identifier; cTAKES – clinical Text Analysis and Knowledge Extraction System; LR – Low/Remission disease activity; MH – Medium/High disease activity; EMR – Electronic Medical Record.

Figure 1 presents the general flow of our document-level disease activity prediction process. As most of the information necessary for assigning a disease activity status is contained in the free text EMR clinical narrative, we used an open source Apache Software Foundation NLP System, the clinical Text Analysis and Knowledge Extraction System (cTAKES) [32], [33], to discover clinical named entity mentions (NEs) such as diseases/disorders, signs/symptoms, anatomical sites, procedures, and medications, along with their UMLS code, negation status, and context. Each EMR note is then represented as a vector of features. The multi-dimensional feature space is reduced using feature selection methods. This pruned feature space is then combined with lab values as retrieved from a relation database within the EMR and used to train and evaluate several classification methods to predict the disease activity label.

### Free Text Features and Feature Selection

In our previous work [27] we tested four sets of features to represent the clinical narrative text: (1) a user-defined list of terms, (2) UMLS CUIs, (3) BOWs, and (4) unigrams or word-CUI bigrams. The user-defined dictionary features (also referred to as “customized dictionary”) are entities hand-picked by human experts (study rheumatologists) through chart review or based on their expertise and professional experience. Customized features are usually small in number but their manual generation is a time-consuming process. In contrast, feature sets 2–4 are generated automatically and could be large in number requiring space reduction. We call set 2–4 features “comprehensive automatic features”. UMLS CUI features are medical entity mentions mapped to a UMLS CUI, e.g. in the example in Figure 1 “no joint pain” is represented as the negation of a UMLS concept with CUI C0003862 (-C0003862). BOW features are unordered collections of words that appear in all notes, ignoring stop words, e.g. the example in Figure 1 has the following alphabetically ordered BOW representation – *has, joint, pain, patient, this*. Word-CUI bigram features are the two-unit sequence of a CUIs and its modifier (if such exists in the text). For example, “severe synovitis” is represented as the bigram “severeC0039103”. If, on the other hand, there is no modifier for “synovitis”, it is represented as a unigram “C0039103”. To reduce the space of the comprehensive automatic features, we devised a feature selection pipeline to select the most informative features which we described in a separate manuscript [27]. Briefly, the three-step feature selection pipeline is composed of a frequency cutoff, Chi-squared [34] feature selection, and the Correlation-based Feature Selection (CFS) [35] that uses the genetic algorithm [36] to search for an optimal feature subset. We selected features which had positive chi-square scores with the class label, ignoring features which had zero chi-square scores with the class label where zero is a natural threshold for un-correlated variables. We used the default setting of the Weka [37] Genetic Algorithm tool: crossover probability as 0.6, mutation probability as 0.033 and population size as 20.

### Lab Values as Features

**Figure 2:**
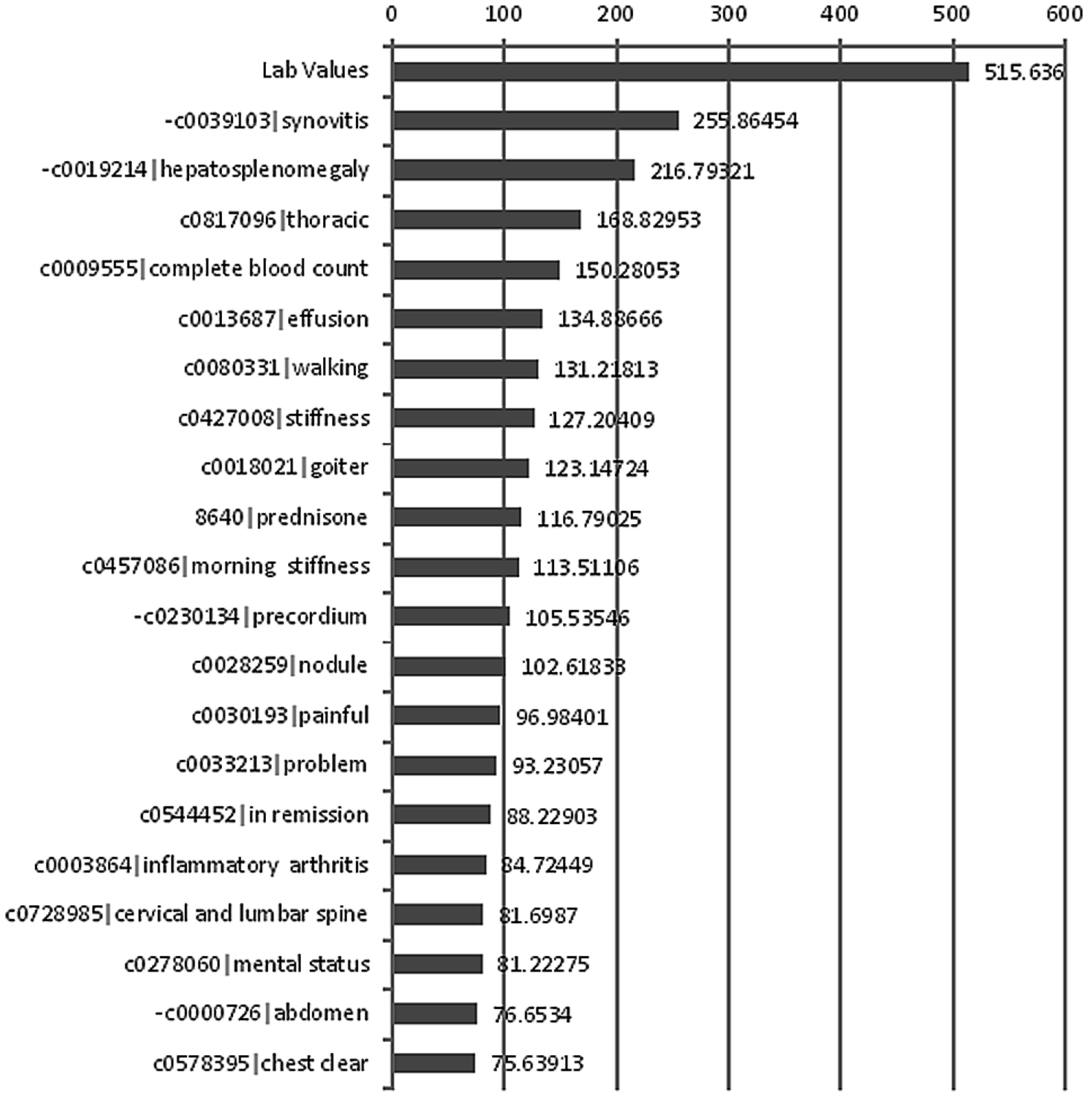
Lab-value and 20 top-ranked CUIs. Their Chi-square values were visualized as bars. Longer bars suggest higher impact. The negative signs “-” before some of the CUIs suggest negation (CUI – Unified Medical Language System Concept Unique Identifier).

The ESR/CRP lab values are stored in a structured database within the EMR and are therefore straightforward to unambiguously extract. We used these values as an additional feature in algorithm development motivated by their relevance in the DAS28 calculation [8], [9], [38]. These lab values were represented as numerical values in our feature space. Figure 2 shows that lab value features (CRP or ESR) are indeed the most informative feature in terms of the Chi-square score.

### Training Selection

**Figure 3:**
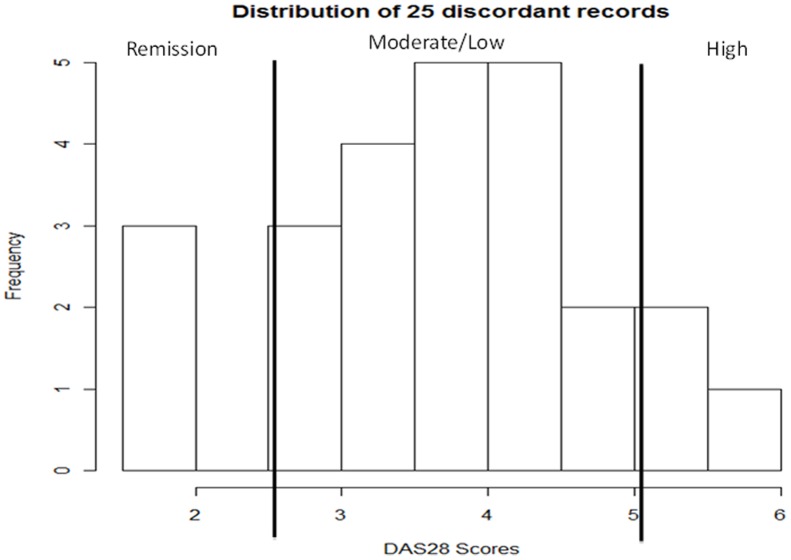
Histogram of DAS28 scores for 25 discordant cases. These discordant cases are between DAS labels and domain expert labels among 93 random samples from the Training Set (the remaining 68 cases were concordant).

In routine practice, it is quite clear when patients have active inflammation or are in complete remission - the extremes on the disease activity spectrum. Not surprisingly, disease activity indices are more accurate for patients with either high or low disease activity [6]. In Collier et al. [6], the physician-predicted disease activity was compared with the calculated DAS. Using the physician-predicted disease activity score as the gold standard, calculated DAS accuracy was greatest for patients with High disease activity (68% accuracy) and those in Remission (75% accuracy), and less accurate for those with Moderate (48%) or Low disease activity (62%) [6]. By studying the IAA between domain expert clinical notes review without available laboratory data and structured DAS-derived labels in the Training Set, we found that the majority of the discrepancies fell in the Moderate and Low disease activity categories (19 cases), while the High and Remission disease activity categories account for only 6 discordances. Figure 3 plots the histogram of the 25 discordant cases.

Therefore, we hypothesized that by removing the Moderate and Low disease activity documents from the Training Set (albeit not from the test set), the classifier can learn concepts that are important in the extreme cases of Remission and High disease activity and avoid terms from the noisier categories of Moderate and Low disease activity. Focusing on these informative terms may not only help classify the extreme cases but also improve the model performance on the middle boundary sections. Beigman and Klebanov [39] showed that adding controversial cases in training could be detrimental to the correct prediction of uncontroversial cases (“hard case bias”). Thus, we compared training on the “extreme” High and Remission labels to training on “all notes” labeled with the aggregate High/Moderate and Low/Remission.

### Classification Method

We used the following classification algorithms in our experiments: Logistic Regression [40], Naïve Bayes [41], Multilayer perceptron [42], Support Vector Machines (SVMs) [43], [44] with linear kernel, SVMs with polynomial kernel, SVMs with Pearson universal kernel [45], and SVMs with Gaussian kernel, all as implemented in Weka [37].

Logistic Regression directly models the posterior class probabilities by applying a logistic sigmoid function on a linear combination of the feature vector. Its parameters are usually estimated by maximum likelihood. Naïve Bayes classifier models the probability of a class given features by applying Bayes' theorem and a strong independence assumption. That is, conditional on the class, the distributions of the feature variables are independent to each other. Multilayer perceptron, also known as the neural network, is a network of multiple layers of nodes in a directed graph. The network can be trained in a supervised fashion by the backward propagation of errors. The information of an input vector will be propagated through the network for output evaluation. SVMs are supervised learning methods that take a set of training data and optimize separations by maximizing the margin between the data categories. SVMs retain input data that lie on the maximum margin hyperplanes as support vectors to define the distinguishing criteria for making predictions on new data. For the data that are not linearly separable in their original space, SVMs have kernel functions that project the data into other feature spaces to achieve better separation.

### Evaluation

Performance is evaluated using standard metrics. *F_1_ score* [31] is the harmonic mean of recall (R) and precision (P): *F_1_* = (2*P*R)/(P+R), where recall is (R = TP/(TP+FN)) and precision is (P = TP/(TP+FP)) where TP is true positives, FN is false negatives, FP is false positives). Area Under the Receiver Operating Characteristic Curve (AUC) [46] is a measure of discrimination that can be viewed as the overall model performance given varied decision boundaries.

To compare the performance, two baselines were used. Baseline 1 is a linear SVMs model; features are BOWs without FS. Baseline 2 is a linear SVMs model; features are BOWs features and lab values. BOWs features are traditionally used as baselines for document classification.

Test Sets were split into 10 folds. Models were tested across all folds for measuring the variance of performance.

## Results

SVMs with a linear kernel deliver the most robust performance especially when Lab values were added as a feature. Detailed results from all experiments can be found in Tables S1, S2, S3, S4, S5. Figures S1, S2, S3 show the top contributing variables with the feature sets and their chi-square values.

**Figure 4:**
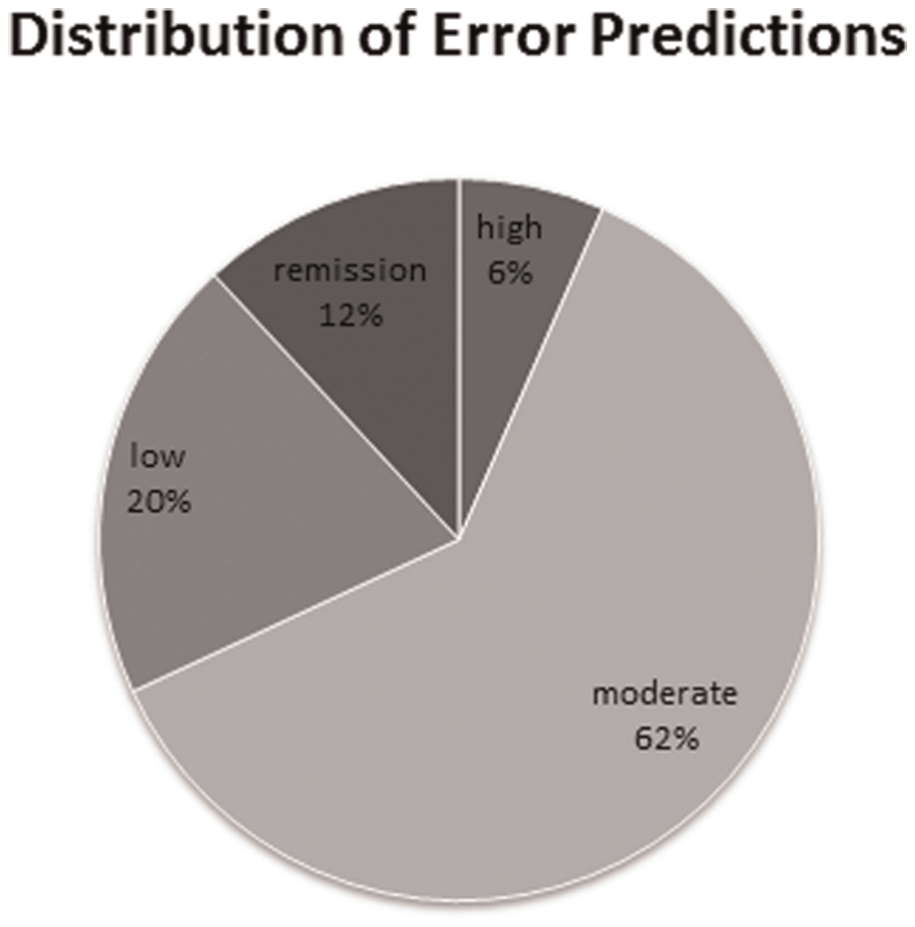
Error analysis of the best performing classifier. Out of 429 misclassified cases (using DAS28 derived dichotomous labels as gold standard), the majority are from the Moderate and Low disease activity categories.

Table 2 shows results on Test Set 1 using a linear-kernel SVM model. The best performing model is the linear-kernel SVM model trained on extremes in the Training Set where the features are the UMLS CUIs after feature selection and ESR/CRP values. Its average 10-fold AUC on the test set evaluation was 0.831, with a standard deviation of 0.0317. Figure 4 shows the distribution of mis-classified cases from the best performing model. The majority of the errors are in the Moderate and Low categories, 62% and 20% respectively. We compared the results from this best performing model with the ones from the other Table 2 models using DeLong test [47] and found it is significantly better (p-values<0.05). The ROC curves of these models are shown in Figure S4.

**Table 2:** Corpus selection effect on Test set 1 using a linear-kernel SVM model.

*Features*	*Training*	*Testing*	*F_1_ score ± σ*	*AUC ± σ*
UMLS CUIs after feature selection and lab values	High and Low Disease Activity labels from Training set	Aggregate High/Moderate and Low/Remission Disease Activity labels from Test Set 1 (10-fold cross-validation)	0.789±0.0445	**0.831±0.0317**
UMLS CUIs after feature selection and lab values	Aggregate High/Moderate and Low/Remission Disease Activity labels from Training Set	Aggregate High/Moderate and Low/Remission Disease Activity labels from Test Set 1 (10-fold cross-validation)	0.747±0.0316	0.810±0.0297
Baseline 1Bag-of-words	Aggregate High/Moderate and Low/Remission Disease Activity labels from Training Set	Aggregate High/Moderate and Low/Remission Disease Activity labels from Test Set 1 (10-fold cross-validation)	0.737±0.0331	0.732±0.0348
Baseline 2Bag-of-words and lab values	Aggregate High/Moderate and Low/Remission Disease Activity labels from Training Set	Aggregate High/Moderate and Low/Remission Disease Activity labels from Test Set 1 (10-fold cross-validation)	0.750±0.0265	0.758±0.0291

For the best performing model in Table 2, we examined the contribution of each feature. The lab value feature is a strong indicator of disease activity. This fact is further supported by its Chi-square value (Figure 2). Table 3 compares the feature contribution given both linear-kernel SVM and Decision Tree [48], a baseline rule-based classifier. It shows that using only the lab value feature gets the majority of classifications correct, even though its effectiveness is not as good as the CUI features. As expected, the best result combines NLP-based features and Lab values.

**Table 3:** Feature contribution.

	*SVM with linear kernel*		*Decision Tree*	
*Features*	*F_1_ score ± σ*	*AUC ± σ*	*F_1_ score ± σ*	*AUC ± σ*
UMLS CUIs	0.740±0.039	0.775±0.036	0.722±0.0602	0.669±0.0641
Lab Values	0.736±0.0393	0.748±0.0300	0.704±0.0419	0.679±0.0337
UMLS CUIs and Lab Values	0.789±0.0445	**0.831±0.0317**	0.74±0.0447	0.714±0.0505

Table 4 shows the results from the portability test. Because the notes in Test Set 2 do not have associated CRP/ESR lab values, these missing values are imputed as the global feature mean by Weka.

**Table 4:** Portability testing.

*Features*	*Training*	*Testing*	*F_1_ score ± σ*	*AUC ± σ*
UMLS CUIs after feature selection and lab values	High and Low Disease Activity labels from Training set	Aggregate High/Moderate and Low/Remission Disease Activity labels from Test Set 2 (10-fold cross-validation)	0.761±0.0553	0.785±0.0599
UMLS CUIs after feature selection and lab values	Aggregate High/Moderate and Low/Remission Disease Activity labels from Training Set	Aggregate High/Moderate and Low/Remission Disease Activity labels from Test Set 2 (10-fold cross-validation)	0.646±0.0863	0.748±0.0944

## Discussion

The best performing disease activity classifier utilizes a representation of the clinical narrative as UMLS CUIs pruned by feature selection and combined with lab values from structured EMR databases. The *F_1_ score* of the best model approaches the human expert agreement. As demonstrated by Collier et al [6] and Figure 3, most of the discrepancies between rheumatologist ratings of disease activity (without knowledge of ESR or CRP lab results) and DAS28 occur in the Moderate and Low categories. We hypothesized that excluding these categories from training (albeit not from testing) would improve discrimination. As expected, results did improve (AUC 0.81 to 0.83 in Table 2). Since IAA between rheumatologists and DAS28 range from 0.81–0.87 when they do not have results available from ESR or CRP, we hypothesized that laboratory test results would be strong predictors of DAS28 categories. We found that adding lab values to the models improved discrimination from 0.78 to 0.83.

**Figure 5:**
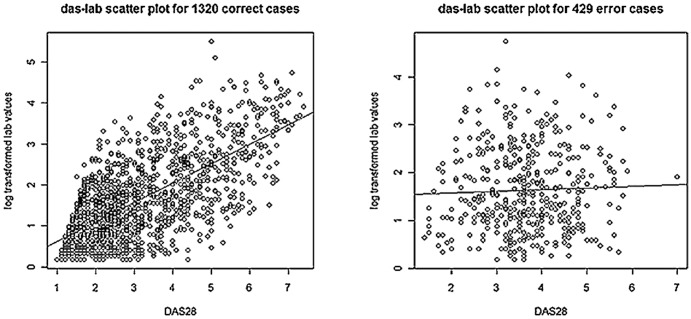
Scatter plot of DAS28 scores and log transformed lab values. (Left) Scatter plot of DAS28 scores and log transformed lab values for 1320 correctly classified notes. (Right) Scatter plot of DAS28 scores and log transformed lab values for 429 misclassified notes. The lines are the regression lines.

Why is the classification of Low and Moderate disease activity by machine learning problematic? By studying the concordance between the DAS28 scores and lab values, we found that these two values are poorly correlated with the Low and Moderate disease activity labels. For the 429 mis-classified cases, the scatter plot between DAS28 and log transformed lab values appears random (Figure 5, right diagram, Spearman: 0.02 [49]). For the 1320 correctly classified cases, the scatter plot (Figure 5, left diagram) shows relatively good correlation (Spearman: 0.63).

**Figure 6:**
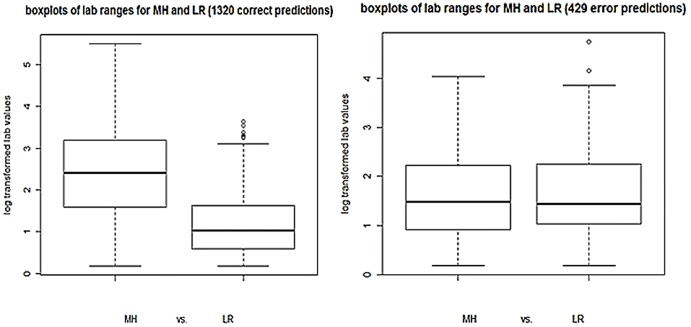
Ranges of lab values. (Left) Range of lab values for Moderate/High (MH) disease activity cases vs. Range of lab values for Low/Remission (LR) disease activity cases among 1320 correctly classified notes. (Right) Range of lab values for Moderate/High (MH) disease activity cases vs. Range of lab values for Low/Remission (LR) disease activity cases among 429 misclassified notes.

It is well known that the ESR and/or CRP values are indicators of disease activity. When the lab value correctly reflects the reality of the patient's disease status, especially for the extreme cases, our model is very accurate. However, if the lab value is less well correlated with clinical aspects of the DAS28 score as in Low and Moderate disease activity documents, the model's performance is strongly influenced by it. The left diagram in Figure 6 points to a lab range corresponding to the different disease activity categories. For the 1320 correctly classified cases, the lab values for the Moderate/High class and the lab values for the Low/Remission class can be separated at 1.5 log value (the first quartile of Moderate/High class meets the third quartile of Low/Remission class at 1.5 log value). However, for the 429 misclassified cases there is no such range pattern (Figure 6, right diagram). Among the 429 errors, given the 1.5 lab boundary, there are 212 notes whose lab values cross the boundary indicating a disease activity category not matching the final DAS28. A possible solution to this problem could be incorporating additional structured codified data, such as the patient self-reported assessment of global health, to help balance the impact of lab values. Another approach is to add a learnable weight for the ESR/CRP feature.

Another possible venue to improve the performance of the classifier is through new feature engineering that incorporates domain knowledge. Asserted relations between relevant entity mentions more precisely represent the details of the clinical events. For example, an asserted *locationOf* relation between a sign/symptom mention and an anatomical site mention such as “swollen wrists” can provide important learnable information for better understanding of the clinical narrative.

Why does the linear-kernel model yield the best performance? There could be several explanations. The lab feature is a dominating feature and by itself has a strong indication of linear separation (i.e. higher lab values indicate higher disease activity levels). For the comprehensive feature sets, we applied chi-square and CFS methods. Chi-square tests and Pearson correlations which the CFS is based on are both not very sensitive to non-linear relationships [50], [51]. Thus the selected features may be dominated by variables that are linearly correlated with the label. We have been working on exploring other statistics that can give balanced measures for both linear and non-linear correlation [28], so that our future feature selection pipeline can include both linearly and non-linearly informative features.

Automatic discovery of document-level disease activity in large EMR datasets is a critical step towards our overarching goal of identifying responders and non-responders to biologic agents for pharmacogenomics research in RA. In the future, we are planning to integrate the automatically generated document-level disease activity labels for the clinical visits with the medication start date to model a general timeline for responders and non-responders.

### Limitations

We made efforts to test the approach for portability on independent previously unseen data (Test Set 1 and Test Set 2). However, our portability tests come from one institution. Expanded testing will port the classifier to a different EMR environment. In order to deploy our disease activity classifier to other institutions, the document filtering criteria (as described in Filtering Criteria S1) would need to be tailored to the specific institution's EMR and then applied to an RA EMR cohort. To maximize the model's performance, each document would benefit from an association with a lab value (either ESR or CRP), though our model can deal with missing ESR/CRP. In addition, we are in the process of porting the methodology to discover disease activity levels for other medical conditions such as Multiple Sclerosis and Inflammatory Bowel Disease.

## Conclusion

In this work we show how within an EMR environment the output of a comprehensive clinical NLP system in combination with lab values stored in structured databases can be used to develop a document-level classifier for the novel phenotype of disease activity in RA. The best performing classifier uses as features lab values and UMLS CUIs after feature selection. The classifier is implemented as a linear kernel SVM to achieve results that are comparable to the human expert agreement. This study is a building block towards the task of identifying responders and non-responders of disease treatments in pharmacogenomics research.

## Supporting Information

Figure S1 **20 top-ranked user-defined customized dictionary features.** Their related Chi-square values were visualized as bars. Longer bars suggest higher impact.
(TIF)

Figure S2 **20 top-ranked unigram and word-CUI bigram features.** Their Chi-square values were visualized as bars. Longer bars suggest higher impact. The negative signs “-” before some of the CUIs suggest negation. A bigram is formatted as “CUI_modifier” or “modifier_CUI”, depending on the order between CUI and its modifier/noun in real text. The concept name of each CUI/RxNorm Code is listed after “|”. If there is no nearby modifier or noun word, the CUI is picked up as a unigram, such as RxNORM “8640” has a preferred term of “prednisone”.
(TIF)

Figure S3 **20 top-ranked word features.** Their related Chi-square values were visualized as bars. Longer bars suggest higher impact. “hapatospleno” is the stemmed form of “hepatosplenomegaly”.
(TIF)

Figure S4 **ROC curves of five models tested on the Test set 1.** From top to bottom: (1) The linear-kernel SVM model trained on High and Remission cases of the Training set, using selected CUI features and lab values; (2) The RBF-kernel SVM model trained on High and Remission cases of the Training set, using selected CUI features and lab values; (3) The linear-kernel SVM model trained on all notes of the Training set, using selected CUI features and lab values; (4) Baseline system 2, which is a linear kernel SVM model on all BOW features with lab values; (5) Baseline system 1, which is a linear kernel SVM model on all BOW features without lab values.
(TIF)

Filtering Criteria S1 The filtering criteria were developed iteratively as we reviewed sets of charts and were applied to the test sets. No filtering criteria were applied to the training set.
(DOCX)

Table S1 Number of features for a user-defined customized dictionary, Unified Medical Language System Concept Unique Identifier (UMLS CUI), Word, and Word_CUI bigram on the Training Set.
(DOCX)

Table S2 Portability test for all classifiers trained on Unified Medical Language System Concept Unique Identifier (UMLS CUI) features: using lab feature vs. no lab features.
(DOCX)

Table S3 Portability test for all classifiers trained on user-defined customized dictionary features: using lab feature vs. no lab features.
(DOCX)

Table S4 Portability test for all classifiers trained on word features: using lab feature vs. no lab features.
(DOCX)

Table S5 Portability test for all classifiers trained on word-CUI bigram features: using lab feature vs. no lab features.
(DOCX)

Table S6 Table of abbreviations.
(DOCX)
