# Supplementary figures and images for: Automatic Prediction of Rheumatoid Arthritis Disease Activity from the Electronic Medical Records
Source: PLoS One. 2013 Aug 16;8(8):e69932. doi: 10.1371/journal.pone.0069932 (PMC3745469; doi:10.1371/journal.pone.0069932)

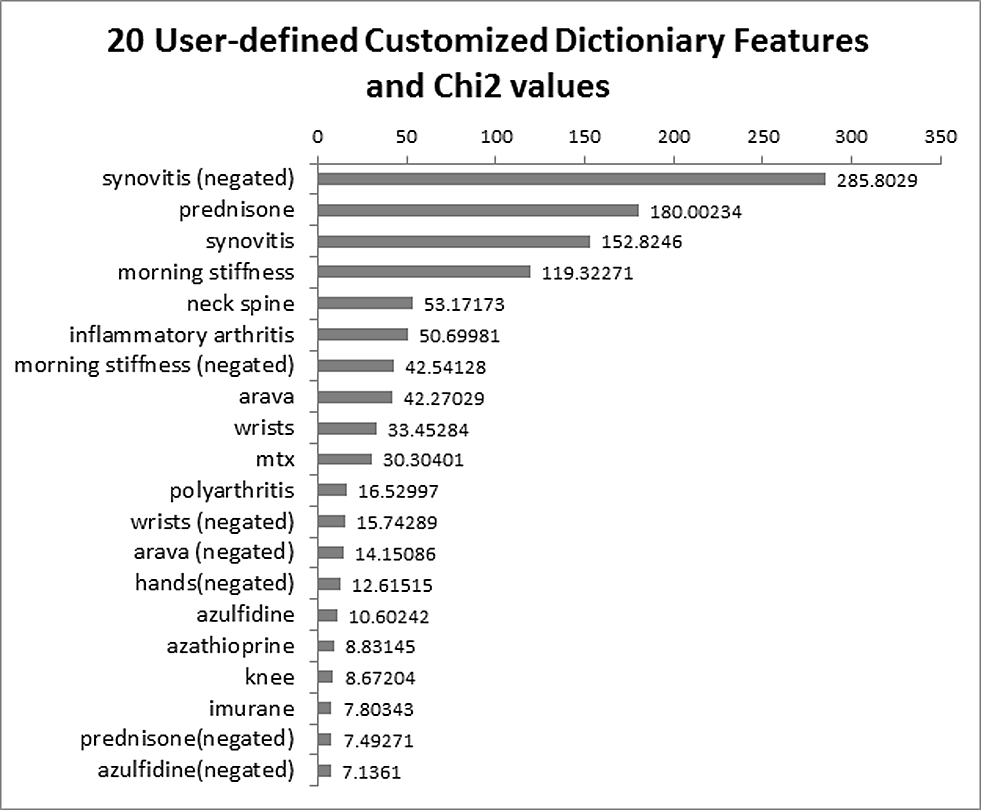

Supplement: Figure S1 — 20 top-ranked user-defined customized dictionary features. Their related Chi-square values were visualized as bars. Longer bars suggest higher impact. (TIF) [file pone.0069932.s001.tif]

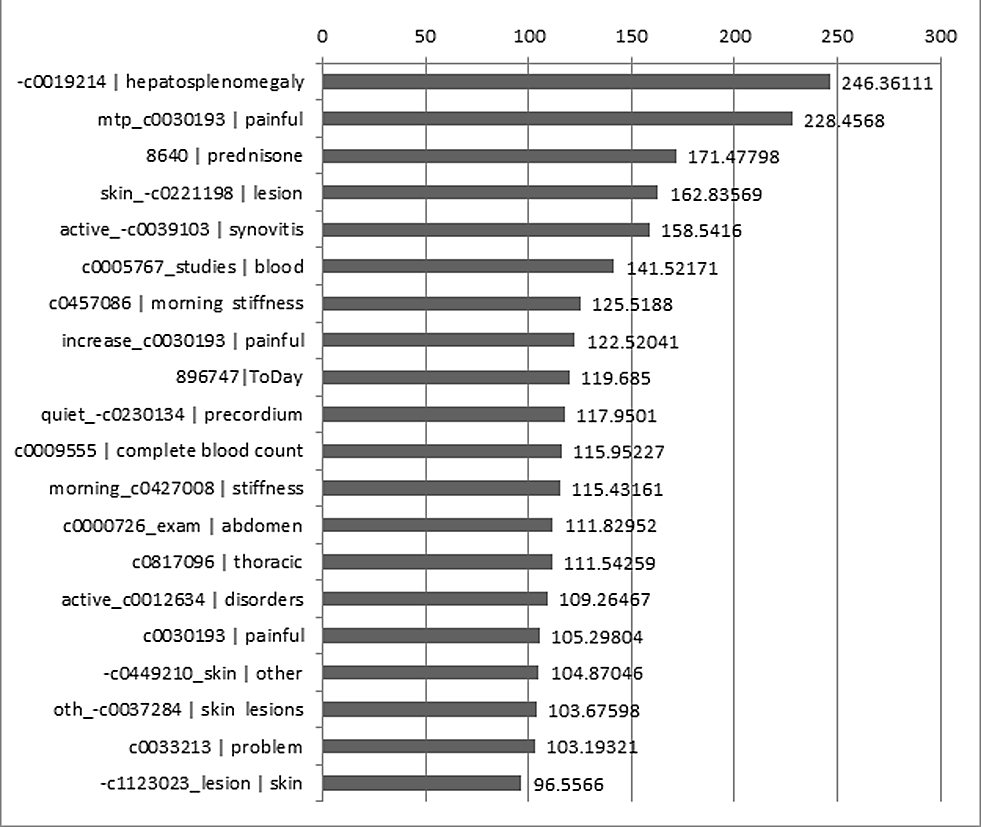

Supplement: Figure S2 — 20 top-ranked unigram and word-CUI bigram features. Their Chi-square values were visualized as bars. Longer bars suggest higher impact. The negative signs “-” before some of the CUIs suggest negation. A bigram is formatted as “CUI_modifier” or “modifier_CUI”, depending on the order between CUI and its modifier/noun in real text. The concept name of each CUI/RxNorm Code is listed after “|”. If there is no nearby modifier or noun word, the CUI is picked up as a unigram, such as RxNORM “8640” has a preferred term of “prednisone”. (TIF) [file pone.0069932.s002.tif]

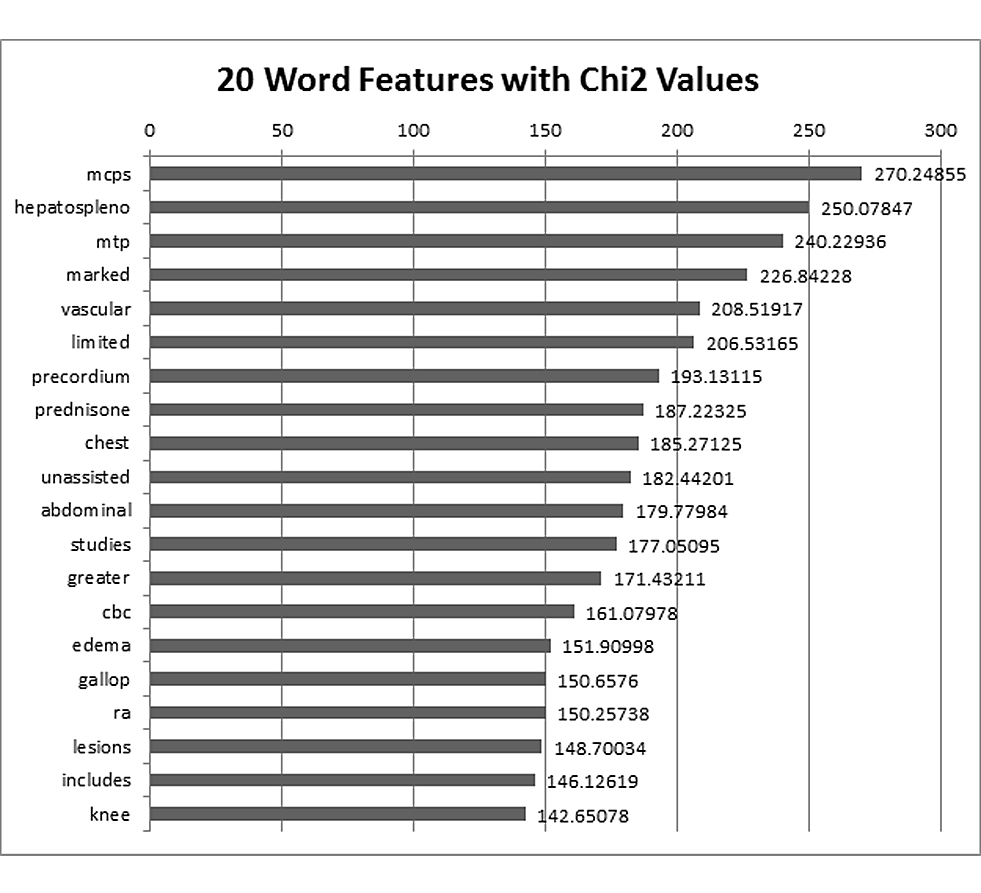

Supplement: Figure S3 — 20 top-ranked word features. Their related Chi-square values were visualized as bars. Longer bars suggest higher impact. “hapatospleno” is the stemmed form of “hepatosplenomegaly”. (TIF) [file pone.0069932.s003.tif]

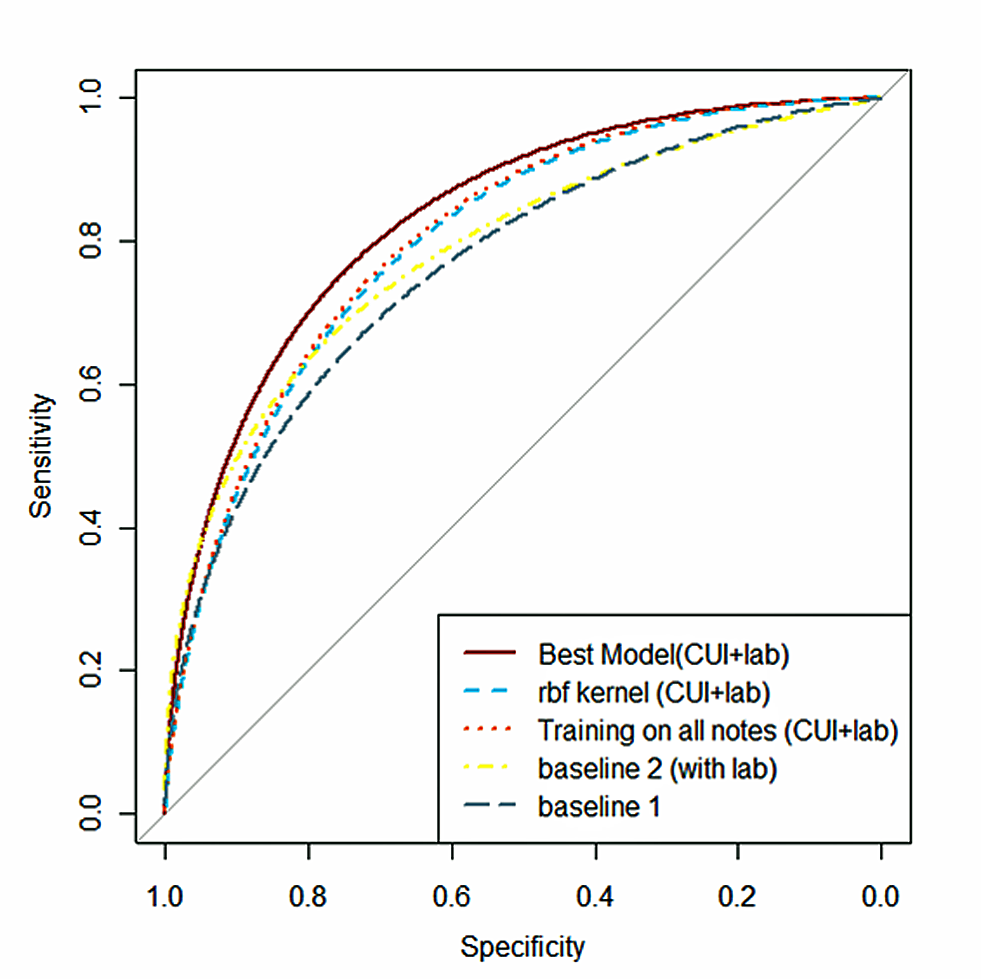

Supplement: Figure S4 — ROC curves of five models tested on the Test set 1. From top to bottom: (1) The linear-kernel SVM model trained on High and Remission cases of the Training set, using selected CUI features and lab values; (2) The RBF-kernel SVM model trained on High and Remission cases of the Training set, using selected CUI features and lab values; (3) The linear-kernel SVM model trained on all notes of the Training set, using selected CUI features and lab values; (4) Baseline system 2, which is a linear kernel SVM model on all BOW features with lab values; (5) Baseline system 1, which is a linear kernel SVM model on all BOW features without lab values. (TIF) [file pone.0069932.s004.tif]
