# Supplementary material for: Automatic Prediction of Rheumatoid Arthritis Disease Activity from the Electronic Medical Records
Source: PLoS One. 2013 Aug 16;8(8):e69932. doi: 10.1371/journal.pone.0069932 (PMC3745469; doi:10.1371/journal.pone.0069932)
Supplement: Filtering Criteria S1 — The filtering criteria were developed iteratively as we reviewed sets of charts and were applied to the test sets. No filtering criteria were applied to the training set. (DOCX) [file pone.0069932.s005.docx]

## Filtering criteria

The filtering criteria were developed iteratively as we reviewed sets of charts and were applied to the test sets. No filtering criteria were applied to the training set.

**Filtering criteria (* indicates a filtering criterion applied to Test Set 1 only)**

- RA according to text/string mentions in combination with Liao et al. algorithm set at 97% specificity
- Rheumatologist
- Anti-TNF treatment text/string mentions or coded data (ever)
- Date of TNF mention > Jan 1, 2000
- *Removed Arthritis Center notes
- *match start date with joint exam date

**Note filters:**

- Exclude Notes < 500 characters
- Keep “rheumatology” notes
- Exclude notes containing the following text strings:

Phone Call

Phone Note

Results Manager Letter

Infusion notes

result_end

To whom this may concern

Preceptor note

68 joints

Infusion room

drug:

Procedure note

test results

telephone call

Information sheet

how it works

how they work

Tubercullin PPD

PPD planted

Pneumovax

*Massachusetts General Hospital

*Rheumatology Associates

*with the fellow

*rheumatology fellow

*drug information

*Infusion record

*consult requested by

*Department of Orthopedic Surgery
