# Supplementary material for: Automatic Prediction of Rheumatoid Arthritis Disease Activity from the Electronic Medical Records
Source: PLoS One. 2013 Aug 16;8(8):e69932. doi: 10.1371/journal.pone.0069932 (PMC3745469; doi:10.1371/journal.pone.0069932)
Supplement: Table S1 — Number of features for a user-defined customized dictionary, Unified Medical Language System Concept Unique Identifier (UMLS CUI), Word, and Word_CUI bigram on the Training Set. (DOCX) [file pone.0069932.s006.docx]

**Table S1. Number of features for a user-defined customized dictionary, Unified Medical Language System Concept Unique Identifier (UMLS CUI), Word, and Word_CUI bigram on the Training Set.**

|  | User-defined Dictionary | UMLS CUI | Word | Unigram or Bigram |
| --- | --- | --- | --- | --- |
| No FS | 173 | 6541 | 13,028 | 21,688 |
| Chi-2 | N/A | 508 | 1,624 | 1,329 |
| Chi2 + CFS | N/A | 211 | 179 | 440 |

Abbreviations: FS: feature selection; CFS: correlation-based feature selection
