# Supplementary material for: Automatic Prediction of Rheumatoid Arthritis Disease Activity from the Electronic Medical Records
Source: PLoS One. 2013 Aug 16;8(8):e69932. doi: 10.1371/journal.pone.0069932 (PMC3745469; doi:10.1371/journal.pone.0069932)
Supplement: Table S2 — Portability test for all classifiers trained on Unified Medical Language System Concept Unique Identifier (UMLS CUI) features: using lab feature vs. no lab features. (DOCX) [file pone.0069932.s007.docx]

**Table S2. Portability test for all classifiers trained on Unified Medical Language System Concept Unique Identifier (UMLS CUI) features: using lab feature vs. no lab features.**

| Classifier | With Lab features  Train on Training Set; Test on Test Set 1 | | | | | Without Lab features  Train on Training Set; Test on Test Set 1 | | | | |
| --- | --- | --- | --- | --- | --- | --- | --- | --- | --- | --- |
|  | TPR | FPR | PPV | F1-score | AUC | TPR | FPR | PPV | F1-score | AUC |
| LR | 0.806 | 0.42 | 0.695 | 0.746 | 0.725 | 0.809 | 0.506 | 0.655 | 0.724 | 0.713 |
| MP | 0.844 | 0.414 | 0.708 | 0.77 | 0.802 | 0.82 | 0.451 | 0.683 | 0.745 | 0.773 |
| NB | 0.702 | 0.369 | 0.693 | 0.697 | 0.726 | 0.764 | 0.57 | 0.614 | 0.681 | 0.698 |
| SMO_line | 0.856 | 0.365 | 0.736 | **0.789** | **0.831** | 0.836 | 0.495 | 0.667 | 0.742 | 0.777 |
| SMO_poly | 0.837 | 0.428 | 0.699 | 0.762 | 0.782 | 0.802 | 0.491 | 0.659 | 0.724 | 0.726 |
| SMO_puk | 0.486 | 0.18 | 0.762 | 0.593 | 0.726 | 0.837 | 0.665 | 0.599 | 0.698 | 0.705 |
| SMO_rbf | 0.819 | 0.403 | 0.707 | 0.759 | 0.803 | 0.803 | 0.498 | 0.657 | 0.723 | 0.762 |

Full FS pipeline was applied. Models were trained on extremes cases, High vs. Remission. “LR”-- Logistic Regression, “MP” -- Multiple perceptron, “NB” -- Naïve Bayes, “SMO_line” -- Support Vector Machine (SVM) with linear kernel, “SMO_poly” -- SVM with polynomial kernel, “SMO_puk” -- SVM with Pearson universal kernel, “SMO_rbf” -- SVM with Gaussian kernel
