# Supplementary material for: Automatic Prediction of Rheumatoid Arthritis Disease Activity from the Electronic Medical Records
Source: PLoS One. 2013 Aug 16;8(8):e69932. doi: 10.1371/journal.pone.0069932 (PMC3745469; doi:10.1371/journal.pone.0069932)
Supplement: Table S3 — Portability test for all classifiers trained on user-defined customized dictionary features: using lab feature vs. no lab features. (DOCX) [file pone.0069932.s008.docx]

**Table S3. Portability test for all classifiers trained on user-defined customized dictionary features: using lab feature vs. no lab features.**

| Classifier | With Lab features  Train on Training Set; Test on Test Set 1 | | | | | Without Lab features  Train on Training Set; Test on Test Set 1 | | | | |
| --- | --- | --- | --- | --- | --- | --- | --- | --- | --- | --- |
|  | TPR | FPR | PPV | F1-score | AUC | TPR | FPR | PPV | F1-score | AUC |
| LR | 0.831 | 0.367 | 0.727 | 0.775 | 0.782 | 0.801 | 0.497 | 0.654 | 0.72 | 0.723 |
| MP | 0.801 | 0.367 | 0.719 | 0.758 | 0.797 | 0.792 | 0.492 | 0.654 | 0.716 | 0.725 |
| NB | 0.785 | 0.301 | 0.754 | 0.769 | 0.823 | 0.755 | 0.398 | 0.69 | 0.721 | 0.756 |
| SMO_line | 0.843 | 0.355 | 0.736 | 0.786 | 0.824 | 0.814 | 0.485 | 0.663 | 0.731 | 0.753 |
| SMO_poly | 0.783 | 0.347 | 0.726 | 0.754 | 0.787 | 0.765 | 0.483 | 0.65 | 0.703 | 0.703 |
| SMO_puk | 0.745 | 0.289 | 0.752 | 0.748 | 0.791 | 0.744 | 0.37 | 0.703 | 0.723 | 0.75 |
| SMO_rbf | 0.803 | 0.34 | 0.735 | 0.767 | 0.809 | 0.801 | 0.436 | 0.683 | 0.737 | 0.766 |

Full FS pipeline was applied. Models were trained on extremes cases, High vs. Remission. “LR”-- Logistic Regression, “MP” -- Multiple perceptron, “NB” -- Naïve Bayes, “SMO_line” -- Support Vector Machine (SVM) with linear kernel, “SMO_poly” -- SVM with polynomial kernel, “SMO_puk” -- SVM with Pearson universal kernel, “SMO_rbf” -- SVM with Gaussian kernel
