# Supplementary material for: Automatic Prediction of Rheumatoid Arthritis Disease Activity from the Electronic Medical Records
Source: PLoS One. 2013 Aug 16;8(8):e69932. doi: 10.1371/journal.pone.0069932 (PMC3745469; doi:10.1371/journal.pone.0069932)
Supplement: Table S4 — Portability test for all classifiers trained on word features: using lab feature vs. no lab features. (DOCX) [file pone.0069932.s009.docx]

**Table S4. Portability test for all classifiers trained on word features: using lab feature vs. no lab features.**

| Classifier | With Lab features  Train on Training Set; Test on Test Set 1 | | | | | Without Lab features  Train on Training Set; Test on Test Set 1 | | | | |
| --- | --- | --- | --- | --- | --- | --- | --- | --- | --- | --- |
|  | TPR | FPR | PPV | F1-score | AUC | TPR | FPR | PPV | F1-score | AUC |
| LR | 0.755 | 0.597 | 0.597 | 0.667 | 0.597 | 0.857 | 0.558 | 0.643 | 0.735 | 0.668 |
| MP | 0.963 | 0.872 | 0.564 | 0.712 | 0.747 | 0.853 | 0.517 | 0.66 | 0.744 | 0.765 |
| NB | 0.739 | 0.473 | 0.647 | 0.69 | 0.685 | 0.742 | 0.537 | 0.619 | 0.675 | 0.667 |
| SMO_line | 0.888 | 0.517 | 0.669 | 0.763 | 0.789 | 0.88 | 0.538 | 0.657 | 0.753 | 0.767 |
| SMO_poly | 0.8 | 0.379 | 0.713 | 0.754 | 0.751 | 0.822 | 0.504 | 0.657 | 0.731 | 0.718 |
| SMO_puk | 0.425 | 0.214 | 0.7 | 0.529 | 0.667 | 0.577 | 0.277 | 0.71 | 0.637 | 0.705 |
| SMO_rbf | 0.859 | 0.518 | 0.661 | 0.747 | 0.783 | 0.83 | 0.531 | 0.647 | 0.727 | 0.754 |

Full FS pipeline was applied. Models were trained on extremes cases, High vs. Remission. “LR”-- Logistic Regression, “MP” -- Multiple perceptron, “NB” -- Naïve Bayes, “SMO_line” -- Support Vector Machine (SVM) with linear kernel, “SMO_poly” -- SVM with polynomial kernel, “SMO_puk” -- SVM with Pearson universal kernel, “SMO_rbf” -- SVM with Gaussian kernel
