# Supplementary material for: Automatic Prediction of Rheumatoid Arthritis Disease Activity from the Electronic Medical Records
Source: PLoS One. 2013 Aug 16;8(8):e69932. doi: 10.1371/journal.pone.0069932 (PMC3745469; doi:10.1371/journal.pone.0069932)
Supplement: Table S5 — Portability test for all classifiers trained on word-CUI bigram features: using lab feature vs. no lab features. (DOCX) [file pone.0069932.s010.docx]

**Table S5. Portability test for all classifiers trained on word-CUI bigram features: using lab feature vs. no lab features.**

| Classifier | With Lab features  Train on Training Set Test on Test Set 1 | | | | | Without Lab features  Train on Training Set Test on Test Set 1 | | | | |
| --- | --- | --- | --- | --- | --- | --- | --- | --- | --- | --- |
|  | TPR | FPR | PPV | F1-score | AUC | TPR | FPR | PPV | F1-score | AUC |
| LR | 0.804 | 0.61 | 0.607 | 0.692 | 0.607 | 0.763 | 0.531 | 0.628 | 0.689 | 0.638 |
| MP | 0.922 | 0.753 | 0.59 | 0.719 | 0.779 | 0.927 | 0.815 | 0.572 | 0.707 | 0.74 |
| NB | 0.791 | 0.536 | 0.634 | 0.704 | 0.737 | 0.71 | 0.504 | 0.623 | 0.664 | 0.673 |
| SMO_line | 0.871 | 0.432 | 0.703 | 0.778 | 0.821 | 0.859 | 0.514 | 0.662 | 0.748 | 0.773 |
| SMO_poly | 0.752 | 0.276 | 0.762 | 0.757 | 0.802 | 0.821 | 0.504 | 0.657 | 0.73 | 0.704 |
| SMO_puk | 0.179 | 0.036 | 0.854 | 0.295 | 0.712 | 0.683 | 0.381 | 0.678 | 0.68 | 0.693 |
| SMO_rbf | 0.826 | 0.403 | 0.706 | 0.761 | 0.82 | 0.832 | 0.483 | 0.669 | 0.742 | 0.761 |

Full FS pipeline was applied. Models were trained on extremes cases, High vs. Remission. “LR”-- Logistic Regression, “MP” -- Multiple perceptron, “NB” -- Naïve Bayes, “SMO_line” -- Support Vector Machine (SVM) with linear kernel, “SMO_poly” -- SVM with polynomial kernel, “SMO_puk” -- SVM with Pearson universal kernel, “SMO_rbf” -- SVM with Gaussian kernel
