# Supplementary material for: Automatic Prediction of Rheumatoid Arthritis Disease Activity from the Electronic Medical Records
Source: PLoS One. 2013 Aug 16;8(8):e69932. doi: 10.1371/journal.pone.0069932 (PMC3745469; doi:10.1371/journal.pone.0069932)
Supplement: Table S6 — Table of abbreviations. (DOCX) [file pone.0069932.s011.docx]

**Table S6. Table of abbreviations**

| **Abbreviations** | **Terms** |
| --- | --- |
| AUC | The Area Under the Receiver Operating Characteristic Curve |
| BOW | Bag-of-words |
| BRASS | Brigham Rheumatoid Arthritis Sequential Study |
| CFS | Correlation-based Feature Selection (CFS) |
| CRP | C-reactive protein level |
| cTAKES | clinical Text Analysis and Knowledge Extraction System |
| CUI | Concept Unique Identifiers |
| DAS | Disease Activity Score |
| DAS28 | Disease Activity Score in 28 joints |
| DMARDs | Disease activity modifying anti-rheumatic drugs |
| EMR | Electronic Medical Records |
| ESR | erythrocyte sedimentation rate |
| F1 | F1 score: harmonic mean of recall (R) and precision (P) |
| FN | False Negatives |
| FP | False Positives |
| FPR | False Positive Rate |
| FS | Feature Selection |
| IAA | inter-annotator agreement |
| LR | Low/Remission |
| MD | Doctor of Medicine |
| MH | Moderate/High |
| ML | Machine Learning |
| NE | Named Entities |
| NLP | Natural Language Processing |
| P | Precision |
| PPV | positive predictive value |
| R | Recall |
| RA | Rheumatoid Arthritis |
| RBF-kernel | Gaussian Kernel (Radial Basis Function kernel) |
| ROC | Receiver Operating Characteristic Curve |
| SVMs | Support Vector Machines |
| TP | True Positives |
| TPR | True Positive Rate |
| UMLS | Unified Medical Language System |
